# Artificial intelligence for pathologic myopia classification based on the META-PM system: a systematic review and meta-analysis

**DOI:** 10.3389/fphys.2026.1909816

**Published:** 2026-08-05

**Authors:** Yuting Hu, Xiaoyan Wang, Yuke Ji, Yuming Chen, Ziqi Liang, Sheng Wu, Mingying Lai, Weihua Yang

**Affiliations:** 1 Department of Ophthalmology and Optometry, Fujian Medical University, Fuzhou, China; 2 Shenzhen Eye Hospital, Jinan University, Shenzhen, China

**Keywords:** artificial intelligence, clinical utilization, meta-analysis, META-PM, pathologic myopia

## Abstract

**Background:**

Artificial intelligence (AI) has shown considerable potential for pathologic myopia (PM) detection, yet its overall diagnostic performance under the META-PM classification framework remains uncertain.

**Methods:**

A comprehensive literature search was conducted across PubMed, Web of Science, IEEE Xplore, and Embase up to February 10, 2026. Studies applying deep learning models for PM classification using the META-PM system were included. Pooled sensitivity, specificity, and hierarchical summary receiver operating characteristic (HSROC) analyses were calculated. Twelve studies were eligible for the systematic review, of which ten were included in the meta-analysis.

**Results:**

For referable PM detection, the pooled sensitivity and specificity were 0.96 (95% CI: 0.93–0.97) and 0.98 (95% CI: 0.96–0.99), respectively. For PM classification, the pooled macro-area under the receiver operating characteristic curve (AUC) reached 0.99 (95% CI: 0.98–1.00), indicating excellent overall diagnostic performance. Fagan nomogram analysis demonstrated favorable post-test probabilities across different clinical scenarios. The meta-regression identified external validation and image input resolution as significant sources of heterogeneity across studies.

**Conclusion:**

Overall, AI demonstrates outstanding performance for automated PM detection and grading under the META-PM framework. Future studies should focus on multicenter external validation, prospective clinical evaluation, and integration with emerging ultra-widefield imaging technologies to facilitate real- world implementation in primary eye-care screening and risk stratification systems.

**Systematic review registration:**

https://www.crd.york.ac.uk/PROSPERO/, identifier CRD420261351134.

## Introduction

Recently, myopia has emerged as a major global public health crisis. Researches indicate that by 2050, the global myopic population will reach approximately 5 billion, with nearly 1 billion individuals affected by high myopia, which translates to a profound socioeconomic burden (Ohno-Matsui et al., 2016; Pan et al., 2025). High myopia is typically characterized by excessive axial elongation, inducing progressive structural alterations in retina, choroid and sclera, ultimately culminating in pathologic myopia (PM) (Ohno-Matsui, 2016). Although the definitive quantitative cutoffs for PM remain an ongoing debate, the associated fundus structural anomalies and subjective visual impairment are undoubtable. PM ranks among the leading causes of irreversible visual impairment and blindness worldwide, particularly in East Asia (Sun et al., 2019; Ye et al., 2022). Longitudinal studies demonstrate that in individuals with existing myopic maculopathy, the five-year risk of progression exceeds 10% (Itoi et al., 2023; Fang et al., 2018). Consequently, early identification, regular monitoring, accurate classification and timely intervention are imperative to prevent irreversible loss of visual acuity.

Recent advances in ophthalmic imaging—including color fundus photography (CFP), optical coherence tomography (OCT), ultra-widefield (UWF) imaging, and 3D magnetic resonance imaging (3DMRI)—have revolutionized the multidimensional assessment of the myopic fundus (Ohno-Matsui et al., 2021; Luo et al., 2025). While the OCT-based ATN system (Atrophy, Traction, and Neovascularization) provides detailed cross-sectional insights, the META-PM classification, primarily based on CFP, remains the most widely adopted framework for large-scale epidemiological screening and primary care due to its cost-effectiveness and accessibility (Ruiz-Medrano et al., 2019; Ohno-Matsui et al., 2015). The META-PM system categorizes myopic maculopathy into five grades (C0 to C4) and defines “Plus” lesions, such as lacquer cracks and choroidal neovascularization. According to the IMI Pathologic Myopia guideline, category 2 (C2: diffuse chorioretinal atrophy) is widely recognized as a pivotal threshold in disease progression, serving as a critical indicator for specialist referral and proactive intervention (Ohno-Matsui et al., 2021).

Artificial intelligence (AI), particularly deep learning (DL), has demonstrated remarkable accuracy and efficiency in automated medical image analysis (Wan et al., 2023; He et al., 2025; Lu et al., 2025; Zhao et al., 2022; Du et al., 2021b). Integrating AI with the META-PM classification system holds immense potential for high-throughput, automated PM screening, thereby optimizing the allocation of limited ophthalmological resources (Yuan et al., 2020; Casazza et al., 2025). Although previous systematic reviews have evaluated the diagnostic accuracy of AI for PM, none has specifically synthesized based on the standardized META-PM framework. Therefore, our study focused on the overall performance in referral and five category classification, aiming to provide a comprehensive review focusing on diagnostic performance, clinical applicability, translational potential, and future development for real-world implementation.

## Methods

### Search strategy and selection criteria

This systematic review and meta-analysis registered with PROSPERO (CRD42061351134) and conducted in strictly accordance with PRISMA guidelines. A comprehensive literature search was performed across four major databases: PubMed, Web of Science (WOS), Embase, and IEEE Xplore, from their inception to February 10, 2026. The search strategy incorporated a combination of MeSH words and free text terms targeting artificial intelligence and pathologic myopia, key search terms was demonstrated in **Supplementary Table 1**. Additionally, the reference lists of included studies and relevant review were manually screened to identify further eligible studies.

Studies were included if they met the following criteria: (1) utilized the META-PM classification for myopic maculopathy; (2) reported detailed architectural information of models; (3) provided sufficient data to construct 2 × 2 contingency tables and reported specific diagnostic metric; and (4) comprehensively described the training dataset. Exclusion criteria comprised reviews, letters, conference abstracts without full texts, and studies lacking sufficient quantitative data for meta-analysis.

All retrieved records were imported into EndNote X9 for deduplication. Two independent reviewers (HYT and WXY) initially screened titles and abstracts, excluding irrelevant articles. Subsequently, full texts of the remaining studies were thoroughly scrutinized.

### Data extraction and quality assessment

Data extraction was independently performed by two reviewers (HYT and WXY). Any discrepancies were resolved through consensus or consultation with a third reviewer (JYK). Extracted data contains three domains: (1) dataset characteristics (database source, imaging device and hardware, input and external validation number, data augmentation, and input size); (2) model characteristics (Deep learning architecture, hyperparameters, preprocessing techniques and interpretability methods); and (3) performance metrics (sensitivity, specificity, accuracy, precision, κ, and AUC). For studies reporting multiple algorithms or input sizes using the same cohort, data from the best-performing model were included. According to the META-PM, Category 2 (diffuse chorioretinal atrophy) or worse was defined as the threshold for “referable” myopic maculopathy, serving as the positive reference standard for constructing the 2 × 2 contingency tables.

The methodological quality and risk of bias were independently evaluated by two reviewers. The QUADAS-2 (Quality Assessment of Diagnostic Accuracy Studies 2) tool was employed to assess the clinical diagnostic design, while the CLAIM (Checklist for Artificial Intelligence in Medical Imaging) guidelines were utilized to rigorously evaluate the quality of the AI modeling and reporting.

### Statistical analysis

Statistical analyses were executed using Review Manager 5.4 (The Cochrane Collaboration) and Stata 18.0. For the binary diagnostic task, a bivariate random-effects model was utilized to calculate the pooled sensitivity, specificity, positive likelihood ratio (LR+), negative likelihood ratio (LR-), and diagnostic odds ratio (DOR) with their corresponding 95% confidence intervals (CIs). A Hierarchical Summary Receiver Operating Characteristic (HSROC) curve was plotted to visually summarize the overarching diagnostic performance. For studies reporting the 5 grades task, a random-effects model was conducted to pool the macro-averaged AUCs. To evaluate the potential clinical applicability of the diagnostic test, a Fagan nomogram was constructed using the pooled positive likelihood ratio (LR+) and negative likelihood ratio (LR−). The nomogram estimates the post-test probability of disease based on a predefined pre-test probability and the diagnostic performance of the test. A pre-test probability was assumed under three clinical conditions, firstly according to the diagnostic result in this meta-analysis, then IMI reported PM prevalence was assumed as prevalence in primary care and the prevalence of PM in high myopia (Ohno-Matsui et al., 2021; Yii et al., 2026). Post-test probabilities following positive and negative test results were calculated to assess the extent to which the test result changes disease probability in clinical practice.

Heterogeneity resulting from the threshold effect was evaluated using the Spearman correlation coefficient between sensitivity and the false positive rate, with P < 0.05 denoting a significant threshold effect. Non-threshold heterogeneity was quantified using the I² statistic, interpreted as limited (0-40%), moderate (40-60%), substantial (60-80%), or considerable (80-100%). Univariate meta-regression and subgroup analyses were subsequently performed to explore potential sources of heterogeneity, incorporating the presence of external validation, dataset size, device type, and image input size. Finally, potential publication bias was assessed utilizing Deeks’ funnel plot.

## Result

### Study selection and characteristics

**Figure 1:**
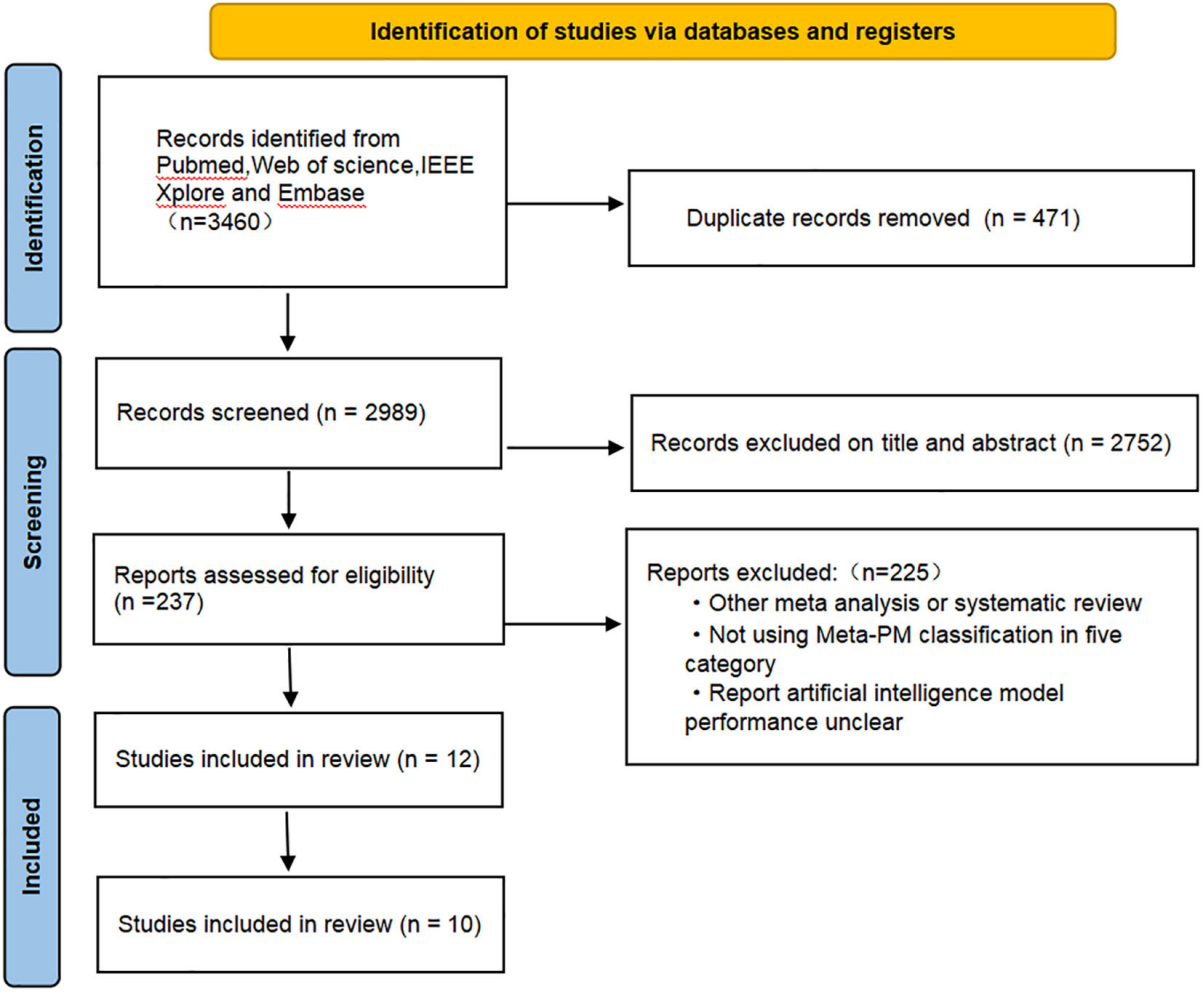
PRISMA 2020 flow diagram of study selection.

A total of 3,460 articles were initially identified through systematic searches in PubMed, Web of Science, IEEE Xplore, and Embase. After removing duplicates, screening titles and abstracts, 237 articles remained for full-text review. Following strict application of the inclusion and exclusion criteria, 12 studies were included in the systematic review,10 provided sufficient data for quantitative meta-analysis (**Figure 1**).

The baseline characteristics of the included studies are summarized in **Table 1**. Overall, 10 studies utilized CFP with a massive input volume of 42,578,867 images, whereas only 2 studies employed UWF imaging. 5 studies incorporated both internal and external validation sets, while the remaining seven relied solely on internal validation. All included studies utilized deep learning for the five categories classification, and 8 studies employed interpretability heatmaps for feature visualization. The detailed model performance on internal and external datasets is presented in **Table 2**.

**Table 1:** Basic characteristic of including research.

Author	Year	Country	Input number	External validation	Data source	Hardware	Device	Input size (pixel)
Yang Liu(Liu et al., 2025)	2025	China	38,922	4449	PSMM dataset	Daytona (P200T) and California (P200DTx)	UWF	224×224;384×384;512×512
Li Lu(Lu et al., 2021a)	2021	China	32010	2059	Eye centers of the First Affiliated Hospital of School of Medicine, Zhejiang University; The First Affiliated Hospital of University of Science and Technology of China; The First Affiliated Hospital of Soochow University	Nonmydriatic retinal cameras (Canon, NIDEK, and Topcon)	CFP	512×512
Li Lu(Lu et al., 2021b)	2021	China	3164	1000	First Affiliated Hospital of School of Medicine, Zhejiang University	Nonmydriatic retinal cameras (Canon)	CFP	512×512
Yun Sun(Sun et al., 2023)	2023	China	1114	400	Beijing TongRen Hospital and PALM dataset	NA	CFP	512×512
Jia Tang(Tang et al., 2022)	2022	China	1395	NA	Peking Union Medical College Hospital (PUMCH), Beijing, China.	Kowa Nonmyd WX-3D (Kowa Company Ltd., Nagoya, Japan) Topcon TRC-50DX (Topcon, Tokyo, Japan) Canon CR-DGi (Canon Inc., Tokyo, Japan)	CFP	224×224; 512×512
Cheng Wan(Wan et al., 2023)	2023	China	1,750	NA	Eye Hospital of Nanjing Medical University	NA	CFP	256×256
Juzhao Zhang(Zhang et al., 2025a)	2025	China	1105	293	Department of Ophthalmology, Shanghai General Hospital	Optos Daytona UWF retinal imaging system	UWF	512×512
Juzhao Zhang(Zhang et al., 2024a)	2024	China	6303	1391	SHMSP	TOPCON DRI Triton	CFP	256×256
Zheming Zhang(Zhang et al., 2024b)	2024	China	2,159	NA	Shenzhen Eye Hospital	NA	CFP	224×224
Bo Zheng(Zheng et al., 2025)	2025	China	7529	NA	Shenzhen Eye Hospital and so on	Topcon (Itabashi-ku, Tokyo, Japan), Canon (Otaku, Tokyo, Japan), and Zeiss (Oberkochen, Germany).	CFP	224×224
Bo Zheng(Zheng et al., 2024)	2023	China	3443	NA	Shenzhen Eye Hospital	NA	CFP	224×224
Shao-Jun Zhu (Zhu et al., 2023)	2023	China	4252	NA	Shenzhen Eye Hospital	NA	CFP	NA

**Table 2:** Model performance in five category tasks.

Author	Modal type	Hyperparameter	Data augment	Preprocessing	Validation strategy	Validation scope	Interpretability	κ	Sensitivity	Specificity	ACC	Precision	AUC
Yang Liu	RealMNet	Yes	Yes	Yes	Hold-out validation	Internal validation and External validation	Yes	0.81	78.52% 94.59% 82.17% 79.87% 77.40%	97.72% 77.58% 92.62% 97.27% 99.23%	94.68%	61.69% 92.44% 63.38% 66.14% 59.79%	97.57%
Li Lu	ResNet18	Yes	Yes	Yes	5-fold cross-validation	Internal validation and External validation	Yes	0.99	97.71% 97.85% 91.34% 96.10% 90.00% 87.03%*	99.91% 97.32% 99.52% 99.58% 99.83% 96.92*	96.74% 87.80%*	99.95% 87.97% 96.47% 89.52% 90.00% 88.34%*	97.9%
Li Lu	Xception	Yes	Yes	Yes	Hold-out validation	Internal validation and External validation	Yes	0.99	98.80% 99.30% 93.70% 95.56% 93.94% 90.34%*	99.65% 99.07% 99.69% 98.78% 99.41% 97.69%*	97.58% 90.57%*	98.86% 98.74% 97.83% 91.49% 93.94% 87.41%*	97.8%
Yun Sun	Resnet50 backbone	Yes	Yes	Yes	Hold-out validation	Internal validation	Yes	0.93	94.32% 92.14% 84.81% 72.55% 85.71%	97.40% 92.86% 96.04% 99.85% 98.65%	89.22%	92.22% 89.27% 85.90% 97.37% 82.35%	99.81%
Jia Tang	ResNet-50	Yes	Yes	Yes	5-fold cross-validation	Internal validation	No	0.93	96.43% 95.56% 95.45% 79.17% 87.50%	98.57% 96.62% 98.00% 99.53% 98.70%	93.7%	90.00% 94.51% 96.55% 95.00% 70.00%	99.80%
Cheng Wan	VOLO-D2 model	Yes	Yes	Yes	Hold-out validation	Internal validation	Yes	0.96	100.00% 96.43% 96.88% 92.31% 100.00%	100.00% 100.00% 98.59% 99.26% 98.10%	96.55%	100.00% 100.00% 93.94% 97.30% 84.21%	98%
Juzhao Zhang	RETFound	Yes	Yes	Yes	Hold-out validation	Internal validation and External validation	No	0.52	44.62% 65.00% 84.95% 62.79% 9.09%	99.12% 91.51% 84.92% 98.39% 99.64%	64.38	93.55% 54.74% 65.83% 67.50% 16.67%	88.6%
Juzhao Zhang	RETFound framework	Yes	Yes	Yes	Hold-out validation	Internal validation and External validation	Yes	0.97	84.74% 93.75% 81.03% 55.88% 93.75% 65.79%*	96.38% 86.07% 98.13% 99.57% 99.66% 90.21%*	90.20% 66.40%*	87.48% 93.34% 77.47% 65.52% 65.22% 72.27%*	98.7%
Zheming Zhang	ResNet50, EfficientNet-B0, ViT, CLIP, RETFound	Yes	Yes	Yes	5-fold cross-validation	Internal validation	Yes	0.98	99.00% 97.83% 97.33% 87.50% 89.74%	98.80% 98.98% 98.32% 99.43% 98.73%	95.37%	96.12% 97.83% 92.41% 97.22% 87.50%	99.5%
Bo Zheng	LTBSoftmax	Yes	Yes	Yes	Hold-out validation	Internal validation	Yes	0.89	100.00% 100.00% 87.11% 83.33% 87.18%	99.93% 99.93% 96.78% 97.57% 99.06%	92.07%	99.69% 99.69% 86.81% 85.60% 85.96%	98.94%
Bo Zheng	EfficientNet	Yes	Yes	Yes	Hold-out validation	Internal validation	NO	0.88	100.00% 93.35% 82.35% 93.00% 96.25%	99.80% 96.35% 95.18% 97.73% 96.69%	88.82%	99.48% 94.39% 80.00% 75.30% 79.38%	98.1%
Shao-Jun Zhu	ALFA-Mix	Yes	Yes	Yes	Hold-out validation	Internal validation	No	0.85	99.21% 95.81% 68.37% 71.22% 62.50%	99.94% 91.82% 95.11% 94.63% 98.12%	94.08%	99.60% 90.81% 75.67% 70.46% 71.43%	No

*demonstrates models’ performance in external validation.

### Study quality and risk of bias assessment

**Figure 2:**
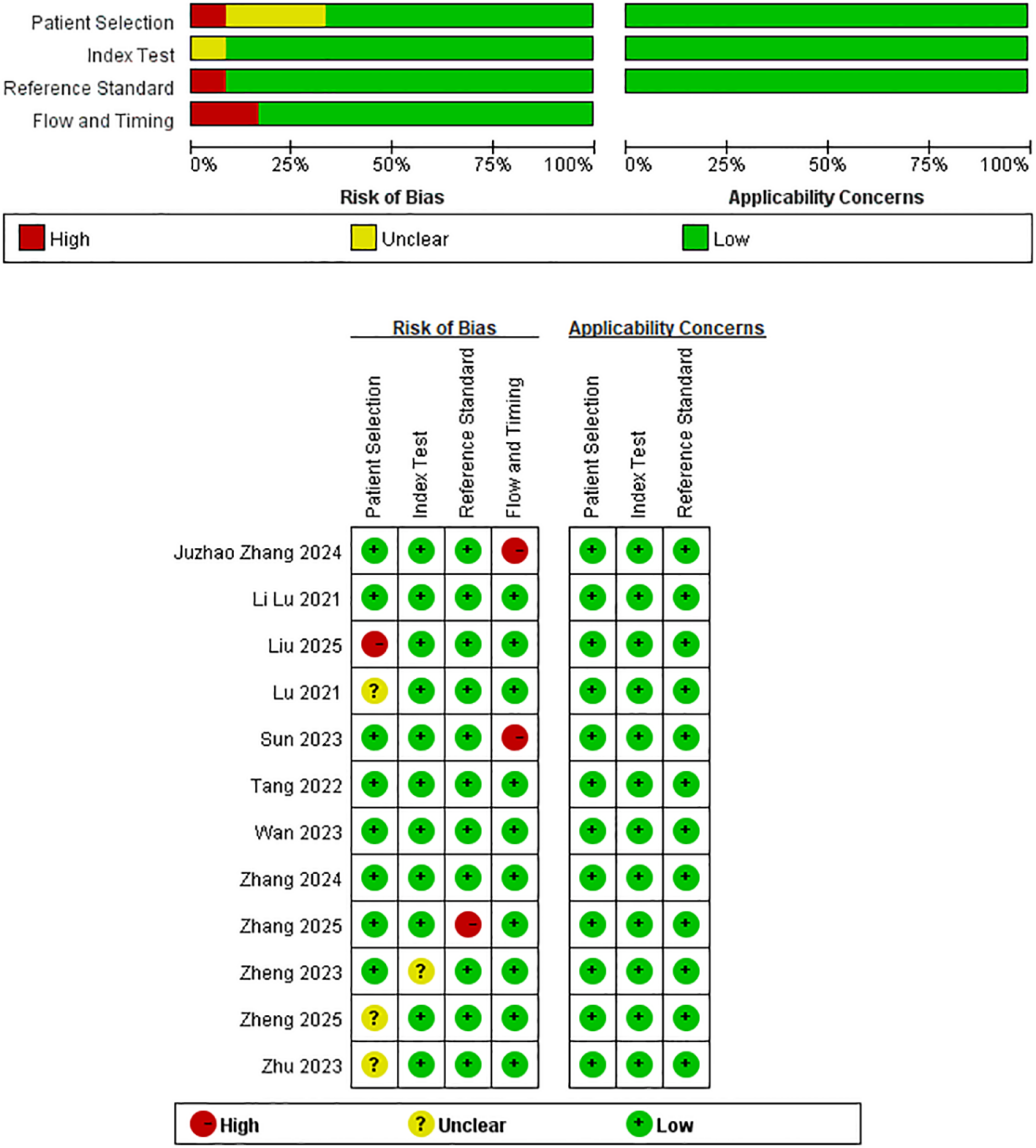
QUADAS-2 for included studies.

**Figure 3:**
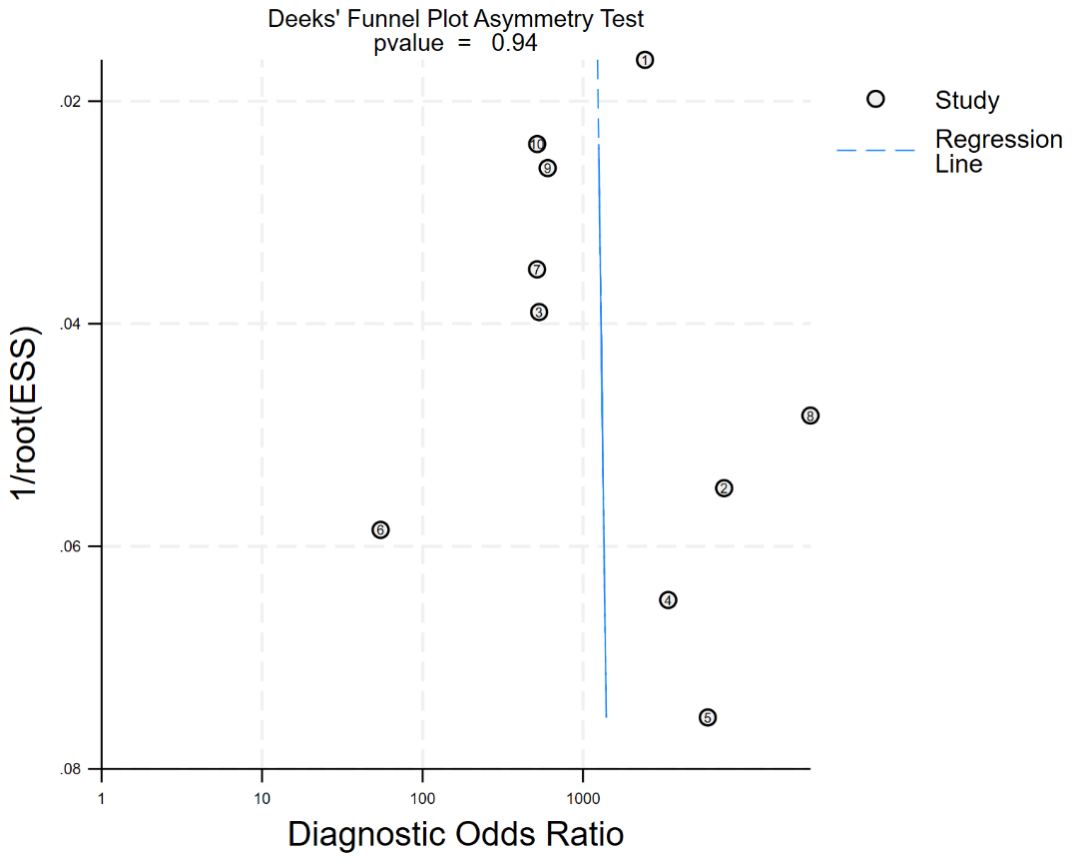
Deeks’ funnel plot for included studies.

Methodological quality and risk of bias were assessed using the QUADAS-2 tool and the CLAIM checklist (**Figure 2**; **Supplementary Table 2**). Overall, 4 studies demonstrated a low risk of bias across all domains, while the remaining 4 exhibited unclear or high risk. Given the retrospective nature of most included studies, details regarding patient selection, flow, and timing were frequently underreported. Applicability concerns were generally low across all domains, indicating that the selected studies highly matched the review question. Furthermore, publication bias was evaluated using Deeks’ funnel plot asymmetry test, which revealed no significant publication bias. The Deeks’ funnel plot is shown in **Figure 3**.

### Diagnostic performance for referable pathologic myopia

**Figure 4:**
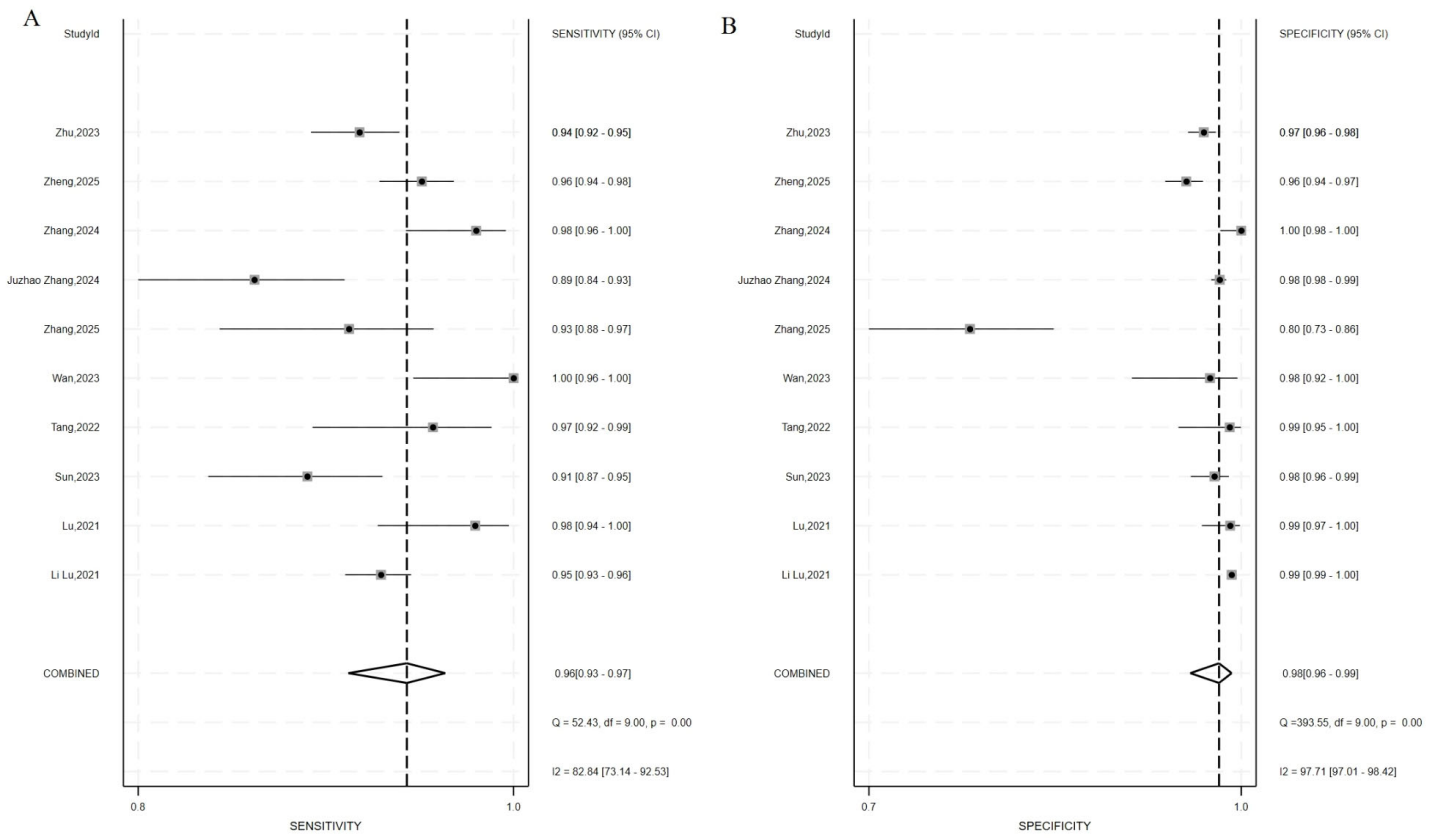
Forest plot of sensitivities **(A)** and specificities **(B)** for main studies.

**Figure 5:**
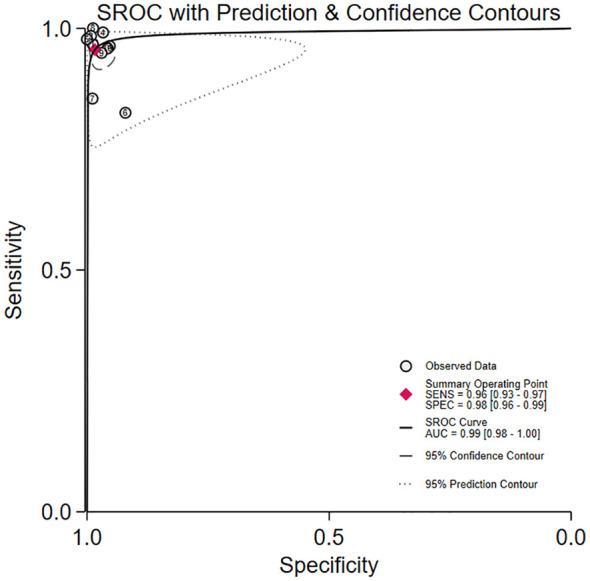
Summary receiver operating characteristic (ROC) plot for main studies.

Based on the META-PM classification, C0 and C1 stages were defined as the non-referable group, while C2 to C4 were categorized as the referable group requiring intervention. **Figure 4** presents the forest plots for sensitivity and specificity across the 10 studies included in the meta-analysis. The reported sensitivity ranged from 0.89 to 1.00, yielding a pooled sensitivity of 0.96 (95% CI: 0.93–0.97) (**Figure 4A**). Similarly, specificity ranged from 0.80 to 1.00, with a pooled specificity of 0.98 (95% CI: 0.96–0.99) (**Figure 4B**). The HSROC curve in **Figure 5**. demonstrated excellent overall diagnostic accuracy, with an area under the curve (AUC) of 0.99 (95% CI: 0.98–1.00). However, considerable heterogeneity was observed in both sensitivity and specificity, and further meta-regression and subgroup analyses are needed to explain.

### Diagnostic performance for the five-category classification

**Figure 6:**
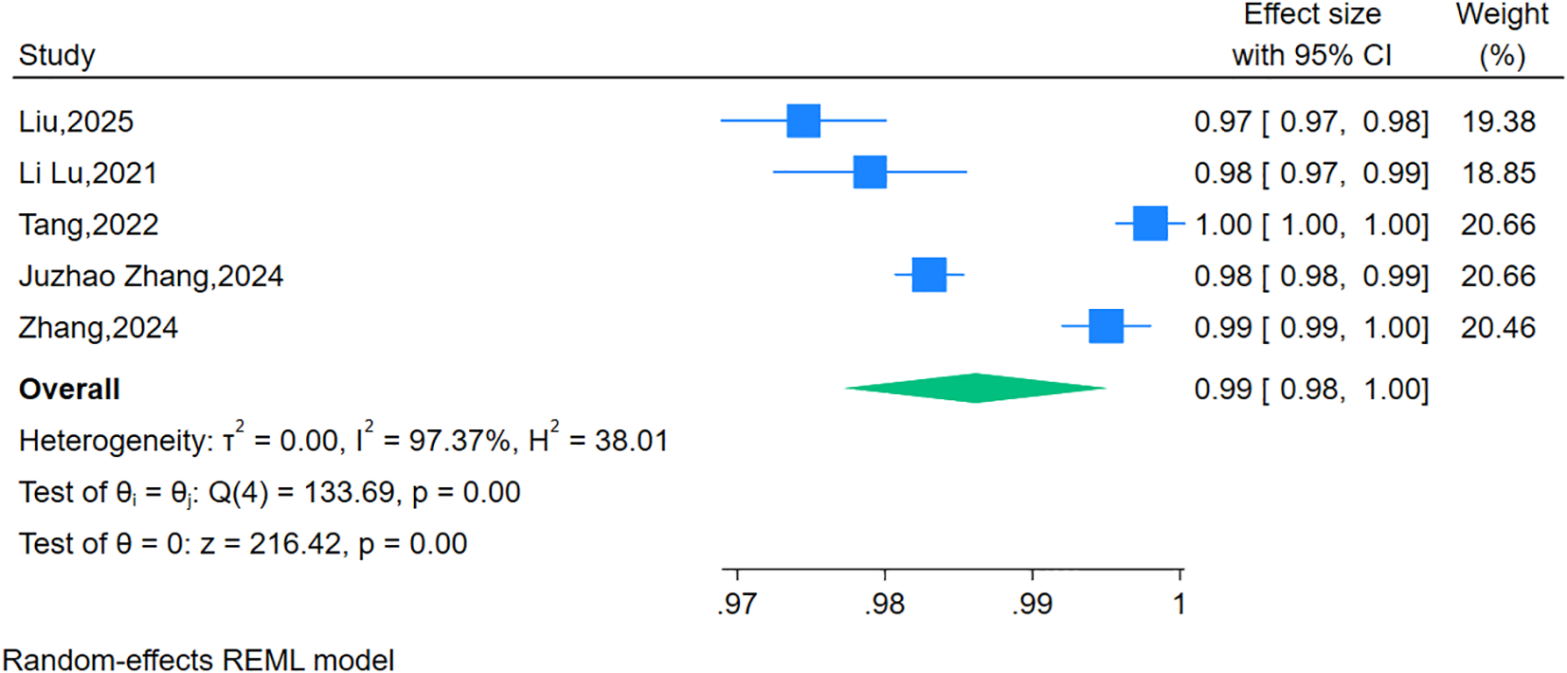
Model 5-category classification performance.

The diagnostic capabilities of the models across the META-PM categories were thoroughly evaluated. The reported sensitivities varied notably across grades: 0.45 to 1.00 for C0, 0.65 to 1.00 for C1, 0.68 to 0.97 for C2, 0.56 to 0.96 for C3, and 0.09 to 1.00 for C4. The corresponding specificities ranged from 0.96 to 1.00 for C0, 0.78 to 1.00 for C1, 0.85 to 0.99 for C2, 0.94 to 0.99 for C3, and 0.90 to 0.99 for C4. The reported AUCs across these categories ranged from 0.87 to 0.99. A separate generic inverse variance meta-analysis was conducted for studies reporting the five-class macro-AUCs with 95% CIs (**Figure 6**), and the AUCs ranged from 0.97 to 1.00. Using a random-effects REML model, the pooled AUC reached 0.99 (95% CI: 0.98–1.00), indicating outstanding discrimination for classification. Nonetheless, substantial heterogeneity was detected.

### Clinical utility of AI in post-test probability assessment

**Figure 7:**
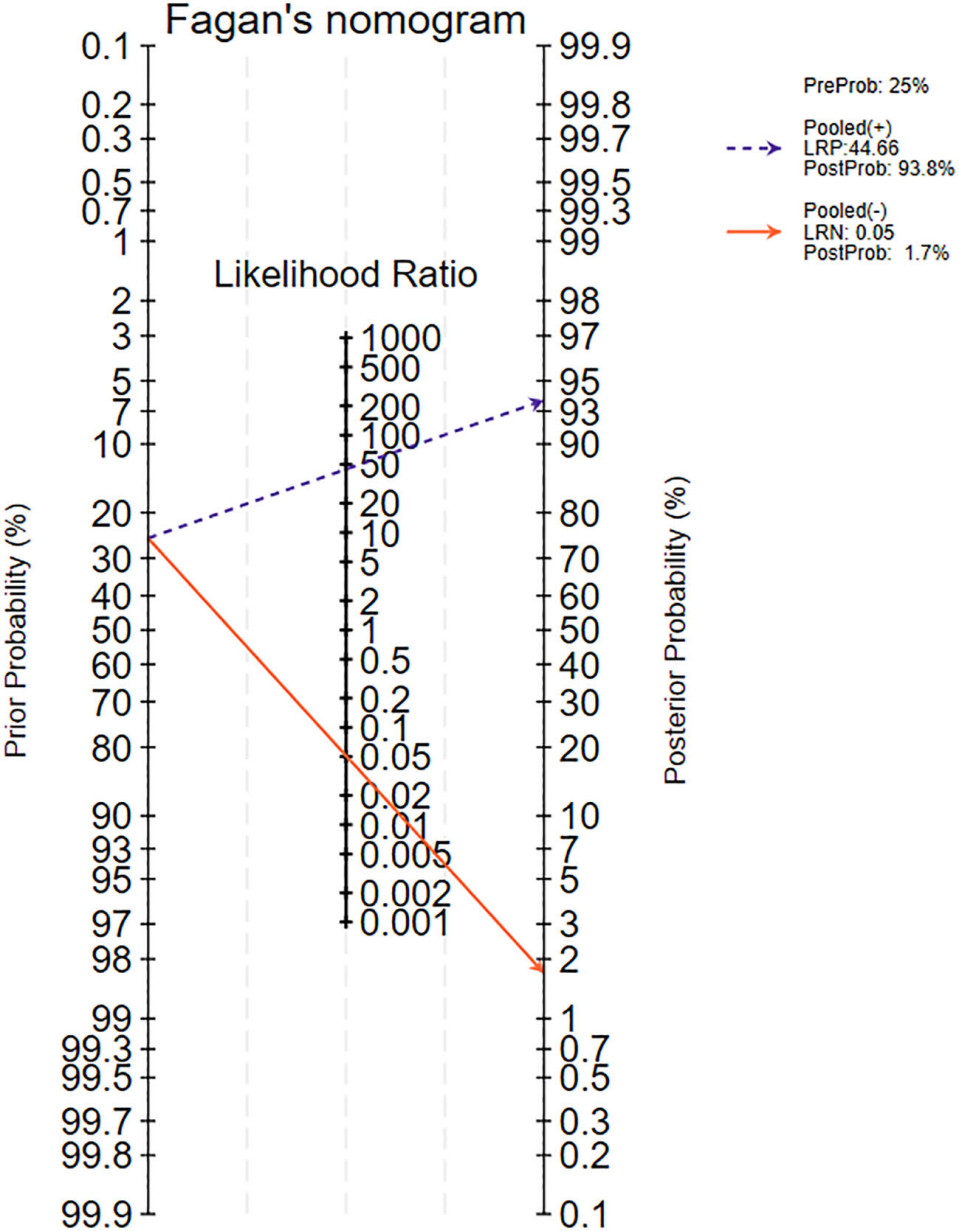
Fagan nomogram for assessing the post-test probability of referable PM using AI under pooled prevalence.

To assess the potential clinical applicability of AI systems for detecting referable PM, Fagan nomogram analysis was performed. We performed our analysis under using the pooled prevalence of referable PM across included studies 25.3%, IMI based PM prevalence 2% and meta-analysis result of PM under high myopia of 47.4% as the pre-test probability. The pooled positive likelihood ratio (LR+) was 44.66, while the pooled negative likelihood ratio (LR−) was 0.05. As illustrated in **Figure 7**, a positive AI result increased the probability of referable PM from 25.3% to 93.8%, whereas a negative AI result reduced the probability to 1.7%. These findings indicate that AI model substantially alter disease probability in identify and reject referable PM. Referable under primary care system with set prevalence of 2% is demonstrated in **Supplementary Figure 1**, a positive result increased the probability of referable PM from 25.3% to 53.6%, whereas a negative result reduced the probability to 0.1%. Referable under high myopia screening condition with set prevalence of 47.4% is demonstrated in **Supplementary Figure 2**, with the positive result of referable PM increased from 25.3% to 98.1%, whereas a negative result reduced the probability to 4.3%.

### Heterogeneity exploration and meta-regression

To determine whether the substantial heterogeneity was driven by the threshold effect, a Spearman correlation test between sensitivity and the false-positive rate was performed. The result (rho = -0.36, P = 0.32) indicated that different diagnostic thresholds were unlikely to be the primary source of heterogeneity. Consequently, univariable meta-regression was conducted using prespecified covariates to explore non-threshold heterogeneity. The results demonstrated that the validation strategy significantly influenced both sensitivity and specificity (P < 0.01). Conversely, dataset size was not significantly associated with either sensitivity (P = 1) or specificity (P = 0.99). Image input resolution did not significantly affect sensitivity (P = 0.53) but was significantly associated with specificity (P < 0.01). Due to the extreme imbalance in device types (9 studies utilizing CFP vs. 1 study utilizing UWF), this variable was summarized descriptively rather than analytically.

### Subgroup analysis

Subgroup analyses were conducted to provide clinically interpretable stratified estimates for the aforementioned covariates. For validation strategy, the pooled sensitivity and specificity were 0.93 (95% CI: 0.90–0.96) and 0.98 (95% CI: 0.94–0.99) in externally validated studies, compared with 0.97 (95% CI: 0.96–0.98) and 0.98 (95% CI: 0.96–0.99) in only internal validated studies. According to input number, studies employing inputs number more than 2662 achieved a pooled sensitivity of 0.95 (95% CI: 0.92–0.96) and a specificity of 0.98 (95% CI: 0.97–0.99), whereas studies less than 2662 demonstrated a sensitivity of 0.97 (95% CI: 0.92–0.98) and a specificity of 0.98 (95% CI: 0.92–0.99). In view of input size, studies with sizes above the median number which is 512 pixels yielded a pooled sensitivity of 0.94 (95% CI: 0.92–0.95) and a specificity of 0.98 (95% CI: 0.94–0.99), compared with 0.97 (95% CI: 0.91–0.99) and 0.98 (95% CI: 0.96–0.99) in studies below the median. Considering devices, studies utilizing CFP, the pooled sensitivity and specificity were 0.93 (95% CI: 0.91–0.96) and 0.96 (95% CI: 0.93–0.98), while the single UWF study reported a sensitivity of 0.53 and a specificity of 0.96 And the results are shown on **Supplementary Table 3** and **Figures 3**–**5**.

## Discussion

The rapid advancement of AI has significantly transformed medical imaging, enabling precise diagnosis, segmentation, and prediction across various ophthalmological conditions, including diabetic retinopathy, glaucoma and so on (Chen et al., 2025; Gong et al., 2025, 2023; Chi et al., 2022; Li et al., 2023a; Xu et al., 2025). Moreover, health economic analysis combined with AI show its great potential in rural and primary medical care (Rajesh et al., 2023; Sun et al., 2024; Wu et al., 2024). Concurrently, the global prevalence of high myopia and PM are increasing at an alarming rate. To standardize PM diagnosis, the META-PM classification was established (Ohno-Matsui et al., 2015). By utilizing highly accessible CFP, this system is particularly beneficial for primary screening in resource limited and rural areas, thereby catalyzing the development of AI models’ development and deployment (Agyekum et al., 2023; de Milliano et al., 2025; Prashar and Tay, 2024). In this systematic review of 12 studies and meta-analysis of 10 studies, we comprehensively evaluated the diagnostic performance of AI models based on the META-PM. Our findings demonstrate immense clinical potential, as the pooled sensitivity and specificity reached 0.96 and 0.98, respectively, and the AUC for the five-category classification task achieved 0.99. Furthermore, the Fagan nomograms showed excellent performance in clinical utilization, indicating that AI can reliably assist in PM detection.

Despite these robust overall metrics, our analysis found a relative reduction in sensitivity for the C2 to C4 classifications, and a marked variability in diagnostic sensitivity for C4 ranged from 0.09 to 1.00 across studies. Several factors may explain this discrepancy. First, the number of C4 is relatively uncommon, resulting in substantial class imbalance in dataset. In addition, the lesions demonstrate considerable morphological heterogeneity in size, shape, and pathological changes, making consistent feature extraction more challenging for model training (Lu et al., 2021a; Zheng et al., 2025). Second, disease development is a continuous process, thus imaging feature, such as lesion size, quantity, and morphology are highly overlap with adjacent classifications, leading to algorithmic “class confusion”. Because missed detection of advanced PM could delay specialist referral and management, future AI systems should prioritize improved recognition of severe disease through larger multicenter datasets, balanced training strategies, and multimodal imaging integration. Furthermore, although C2 or worse is widely accepted as the threshold for referable PM in META-PM classification, referral decisions in clinical practice are often individualized, patients with C1, particularly those with rapid axial elongation, young age, progressive refractive change, may also require closer surveillance and referral. Since all included studies adopted the C2 threshold, future model should incorporate multimodal clinical information beyond fundus grading alone to support personalized referral strategies.

Beyond conventional measures of diagnostic performance, Fagan nomogram analysis was performed to evaluate the potential clinical utility of PM screening. Under the pooled prevalence and likelihood ratios derived from the included studies, community screening, high-myopia clinics conditions, AI systems demonstrated substantial probability modifying capability for future real-world utility. With the increasing global prevalence of myopia, PM has become a major cause of irreversible visual impairment (Du et al., 2021a; Ren et al., 2023). However, limited access to retinal specialists in primary care and rural condition hinder timely identification and referral of high-risk individuals (Mansour et al., 2021). Given its low cost, ease of acquisition, and wide accessibility, CFP remains one of the most practical hardware devices for large-scale screening. The integration of AI with CFP may facilitate rapid and standardized screening, improve referral accuracy, and optimize healthcare resource allocation. Previous studies have demonstrated favorable cost-effectiveness and health-economic benefits of AI-based screening programs for diabetic retinopathy and glaucoma (Huang et al., 2022; Xie et al., 2020; Lin et al., 2023; Xiao et al., 2021). Although similar evidence for PM remains limited, the substantial burden associated with long-term management and vision-threatening complications suggests that AI assisted screening may reduce disease related burden through earlier detection and intervention. Furthermore, multi-disease screening model may play an important role in the future, generating additional clinical and socioeconomic benefits (Liu et al., 2023).

According to the high I², we develop Spearman correlation analysis and exclude threshold effect. To explain the heterogeneity, we include validation strategy and image parameter and device into account. The subgroup meta-analysis demonstrates that external validation and image resolution is significant with diagnosis performance. Not only robust performance, generalization is important for model to fit real world medical treatment in different device and public (Zhang et al., 2025b). Additionally, our findings emphasize that higher image resolution is crucial for optimal feature extraction and precise classification. Regarding of imaging modalities, traditional CFP is limited to a 45°field of view, which restricts the detection of peripheral ocular characteristics. With the wider field imaging devices such as UWF and ultra-field OCT, peripheral defects such as posterior staphylomas can be better visualized, which is vital for ascertaining the true prevalence of PM and reducing the global burden of blindness (Ohno-Matsui, 2016; Zhang et al., 2025a; Liu et al., 2025; Li et al., 2023b). However, while UWF offers a broader perspective, its pseudocolor and peripheral distortion pose classification challenges for algorithms. Therefore, future device development and algorithm refinement must focus on integrating higher resolution, wider fields of view, and realistic color representation to achieve more targeted and accurate classifications.

Despite the promising diagnostic performance demonstrated in this review, AI systems for PM should be translated into clinical practice through a stepwise implementation pathway. First, multicenter prospective studies are needed to validate model robustness across different ethnic populations, imaging devices, and healthcare settings. Second, standardized image acquisition protocols and image quality should be established to ensure consistent and reliable input data. Third, AI models should be integrated into teleophthalmology platforms and routine clinical workflows to provide automated risk stratification and referral recommendations. Importantly, ophthalmologists should remain responsible for reviewing the AI outputs and making final decisions. Such a human–AI collaborative framework may represent the most feasible strategy for translating AI-assisted PM screening into real-world clinical applications.

To our knowledge, this is the first study to systematically evaluate the diagnostic and classification performance of AI models based on META-PM system. Despite robust performance, several limitations must be acknowledged. First, substantial heterogeneity remained despite the meta-regression analysis, including methodological and clinical differences in patient populations, image annotation, model architectures couldn’t be further analyzed because these variables were insufficiently reported in the included studies. Furthermore, the relatively small number of eligible studies limited the statistical power of the meta-regression, preventing a more comprehensive exploration of these potential sources of heterogeneity. Second, most included studies were retrospective and based predominantly on Chinese populations, with images pre-selected by clinicians. Consequently, the distribution of disease categories may not accurately reflect real-world populations, limiting the generalizability of the findings. Third, with the advancement in imaging technology for myopia assessment, particularly UWF and ultra-widefield CFP, evidences regarding the application of the META-PM framework and AI algorithms across these hardware remains limited. Future studies should therefore investigate the robustness and generalizability of AI models across different imaging platforms.

## Conclusion

In conclusion, while artificial intelligence has demonstrated exceptional diagnostic accuracy for PM, its successful clinical translation requires further robust evidence. To bridge the gap between algorithmic development and real-world application, future research must prioritize multicenter external validation, prospective study designs, and the exploration of novel imaging modalities such as UWF imaging. Fulfilling these prerequisites will thoroughly verify the reliability of these models across diverse clinical settings, ultimately facilitating their integration into early screening and risk stratification systems within primary healthcare.

## Data Availability

The original contributions presented in the study are included in the article/**Supplementary Material**. Further inquiries can be directed to the corresponding authors.
