# Supplementary material for: Artificial intelligence for pathologic myopia classification based on the META-PM system: a systematic review and meta-analysis
Source: Front Physiol. 2026 Aug 5;17:1909816. doi: 10.3389/fphys.2026.1909816 (PMC13485559; doi:10.3389/fphys.2026.1909816)
Supplement: Supplementary file 1 [file DataSheet1.pdf]

Table 1. Search strategy

Search item	Related mesh words
Artificial intelligence	artificial intelligence OR machine learning OR deep learning OR transfer learning OR neural network OR robot OR supervised Learning OR unsupervised Learning OR computer vision system OR computational intelligence OR evolutionary computation OR ensemble learning OR reinforcement learning OR large language model OR LLM OR large model
Pathologic myopia	pathological myopia OR pathologic myopia OR high myopia OR myopic maculopathy OR progressive myopia OR refractive error OR myopia

Table 2. Checklist for Artificial Intelligence in Medical Imaging (CLAIM) result in including studies

Study	Title	Abstract	Introduction		Methods																														Results						Discussion	Other Information		
	1	2	3	4	5	6	7	8	9	10	11	12	13	14	15	16	17	18	19	20	21	22	23	24	25	26	27	28	29	30	31	32	33	34	35	36	37	38	39	40	41	42	43	44
**Liu 2025**	Y	Y	Y	Y	Y	Y	Y	Y	Y	Y	Y	NA	Y	Y	Y	Y	Y	Y	Y	Y	Y	Y	Y	Y	Y	Y	NA	Y	Y	Y	Y	Y	Y	NA	Y	Y	Y	Y	Y	Y	Y	Y	Y	Y
**LiLu 2021**	Y	Y	Y	Y	Y	Y	Y	Y	Y	Y	Y	NA	Y	Y	Y	Y	Y	Y	Y	Y	Y	Y	Y	Y	Y	Y	NA	Y	Y	Y	N	Y	Y	NA	Y	Y	N	Y	Y	Y	Y	Y	Y	Y
**Lu 2021**	Y	Y	Y	Y	Y	Y	Y	Y	Y	Y	Y	NA	Y	Y	Y	Y	Y	Y	Y	Y	Y	Y	Y	Y	Y	Y	NA	Y	Y	Y	N	Y	Y	NA	Y	Y	N	Y	Y	Y	Y	Y	Y	Y
**Sun 2023**	Y	Y	Y	Y	Y	Y	Y	Y	N	Y	N	NA	Y	Y	Y	Y	Y	Y	N	Y	Y	Y	Y	Y	Y	Y	NA	Y	Y	N	Y	Y	Y	NA	N	N	Y	Y	Y	Y	Y	Y	Y	Y
**Tang 2022**	Y	Y	Y	Y	Y	Y	Y	Y	Y	Y	Y	NA	Y	Y	Y	Y	Y	Y	Y	Y	Y	Y	Y	Y	Y	Y	NA	Y	Y	Y	N	Y	N	NA	Y	Y	N	Y	Y	Y	Y	Y	Y	Y
**Wan 2023**	Y	Y	Y	Y	Y	Y	Y	Y	Y	Y	Y	NA	Y	Y	Y	Y	Y	Y	N	Y	Y	Y	Y	Y	Y	Y	NA	Y	Y	Y	N	Y	N	NA	Y	N	Y	Y	Y	Y	Y	Y	Y	Y
**Zhang 2025**	Y	Y	Y	Y	Y	Y	Y	Y	Y	Y	Y	NA	Y	Y	Y	Y	Y	Y	N	Y	Y	Y	Y	Y	Y	Y	NA	Y	Y	Y	N	Y	Y	NA	Y	Y	N	Y	Y	Y	Y	Y	Y	Y
**Junzhao Zhang 2024**	Y	Y	Y	Y	Y	Y	Y	Y	N	Y	Y	NA	Y	Y	Y	Y	Y	Y	Y	Y	Y	Y	Y	Y	Y	Y	NA	Y	Y	N	Y	Y	Y	NA	Y	Y	Y	Y	Y	Y	Y	Y	Y	Y
**Zhang 2024**	Y	Y	Y	Y	Y	Y	Y	Y	Y	Y	Y	NA	Y	Y	Y	Y	Y	Y	N	Y	Y	Y	Y	Y	Y	Y	NA	Y	Y	N	Y	Y	N	NA	Y	N	Y	Y	Y	Y	Y	Y	Y	Y

**Zheng 2025**	Y	Y	Y	Y	Y	Y	Y	Y	Y	Y	Y	NA	Y	Y	Y	Y	Y	Y	Y	Y	Y	Y	Y	Y	Y	Y	NA	Y	Y	Y	Y	Y	Y	NA	Y	Y	Y	Y	Y	Y	Y	Y	Y	Y
**Zheng 2023**	Y	Y	Y	Y	Y	Y	Y	N	Y	Y	Y	NA	Y	Y	Y	Y	Y	Y	N	Y	Y	Y	Y	Y	Y	Y	NA	Y	Y	N	Y	Y	N	NA	Y	N	Y	Y	Y	Y	Y	Y	Y	Y
**Zhu 2023**	Y	Y	Y	Y	Y	Y	Y	Y	Y	Y	Y	NA	Y	Y	Y	Y	Y	N	Y	Y	Y	Y	Y	Y	Y	Y	NA	Y	Y	N	Y	Y	N	NA	Y	N	Y	Y	Y	Y	Y	Y	Y	Y

Table 3.Subgroup analysis for the AI model performance

Subgroup	Details	No. of study	SEN(95%CI)	SPE(95%CI)	DOR(95%CI)	LR+(95%CI)	LR-(95%CI)	1/LR-(95%CI)
Validation strategy	I+E	5	0.93 (0.90-0.96)	0.98 (0.94-0.99)	683.72 (172.87-2704.09)	45.73 (14.04-148.4)	0.07 (0.05-0.10)	14.95 (10.12-22.09)
	I	5	0.97 (0.93-0.98)	0.98 (0.96-0.99)	2137.62 (507.45-9004.73)	63.68 (23.46-172.87)	0.03 (0.15-0.06)	33.57 (17.16-65.65)
Resolution	≥512	6	0.94 (0.92-0.95)	0.98 (0.94-0.99)	925.59 (195.18-4389.22)	55.38 (14.12-217.13)	0.06 (0.05-0.08)	16.71 (12.81-21.81)
	＜512	4	0.97 (0.91-0.99)	0.99 0.96-0.99)	2372.51 (392.62-14336.45)	66.60 (22.34-198.53)	0.03 (0.01-0.09)	35.62 (10.98-115.61)
Input number	≥2662	5	0.94 (0.92-0.96)	0.98 (0.97-0.99)	1038.66 (499.07-2161.63)	56.62 (30.89-103.77)	0.05 (0.37-0.08)	18.35 (12.33-27.30)
	＜2662	5	0.97 (0.92-0.98)	0.98 (0.92-0.99)	1827.22 (190.82-17497.4)	63.68 (11.14-363.86)	0.03 (0.02-0.08)	28.70 (12.55-65.62)
Imaging device	CFP	9	0.96 (0.93-0.98)	0.98 (0.97-0.99)	1733.21 (707.38-4246.69)	71.68 (39.79-129.12)	0.04 (0.02-0.07)	24.18 (14.52-40.25)
	UWF	1	0.53	0.95				

*I+E demonstrate validation strategy including internal and external validation

I demonstrate validation strategy only include internal validation

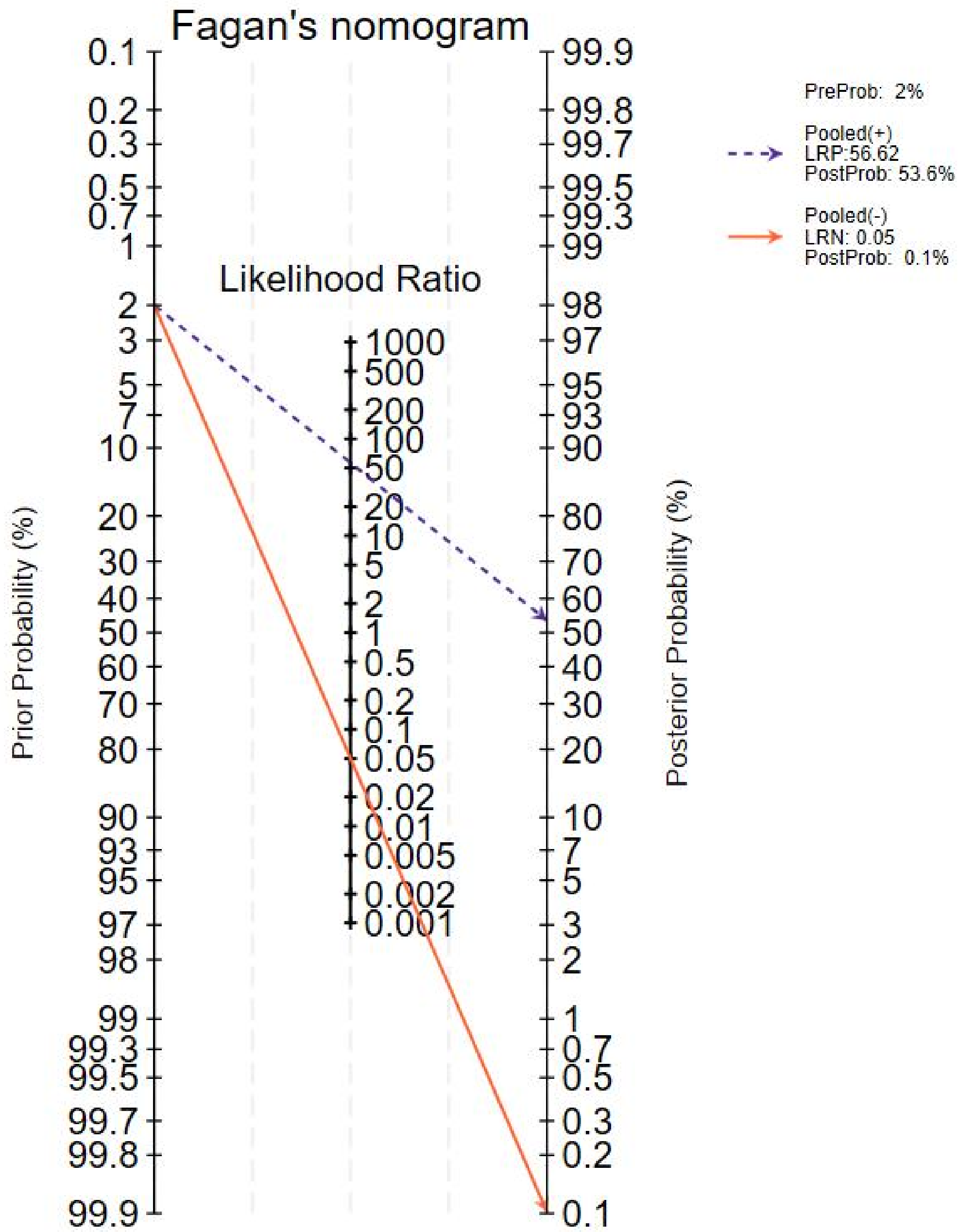

Fig 1. Fagan nomogram for assessing the post-test probability of referable pathologic myopia using AI under primary care

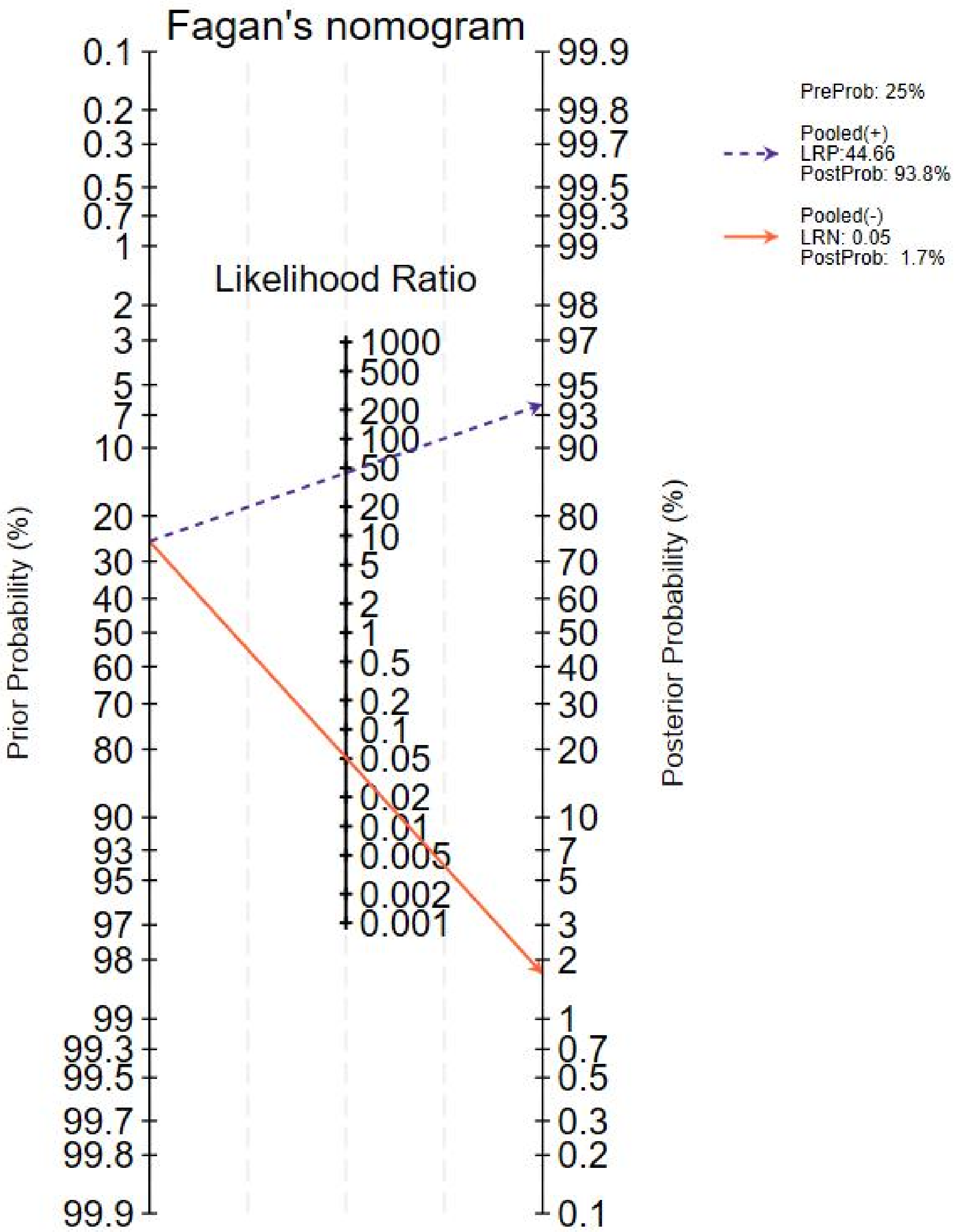

Fig 2. Fagan nomogram for assessing the post-test probability of referable pathologic myopia using AI under high myopia condition

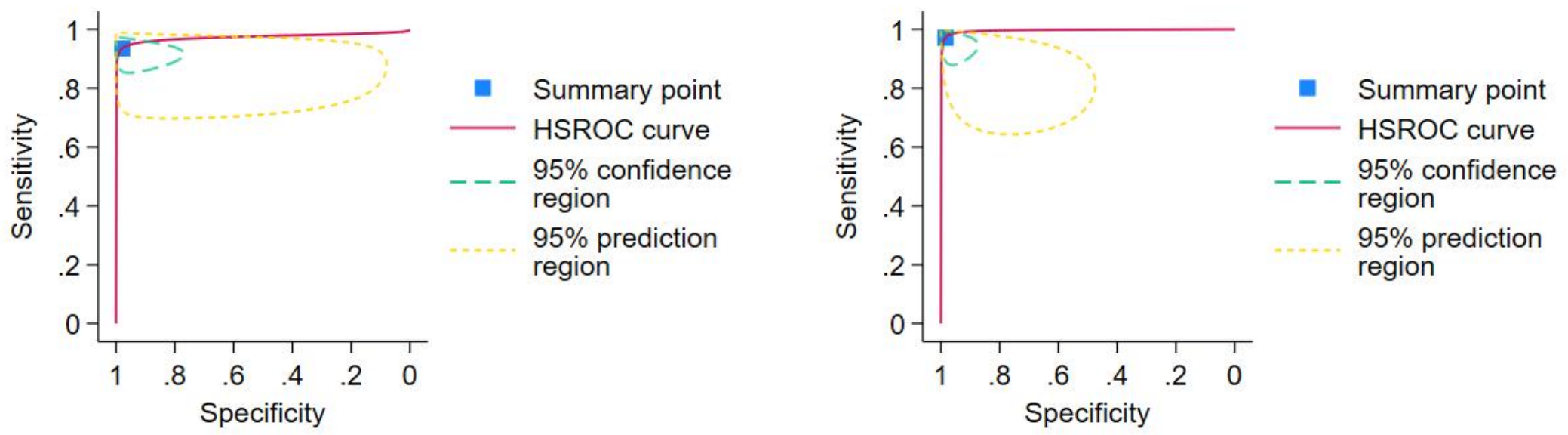

Fig 3 HSROC curve of Validation strategy in internal and external validation and only internal validation

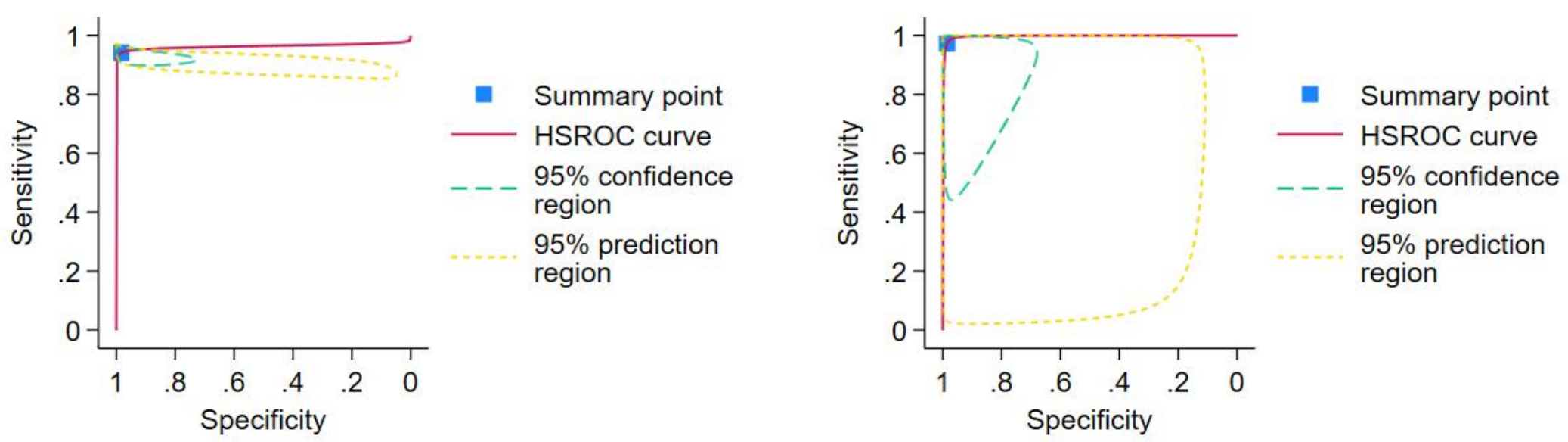

Fig 4 HSROC curve of Resolution in ≥512 pixel group and ＜512 pixel group

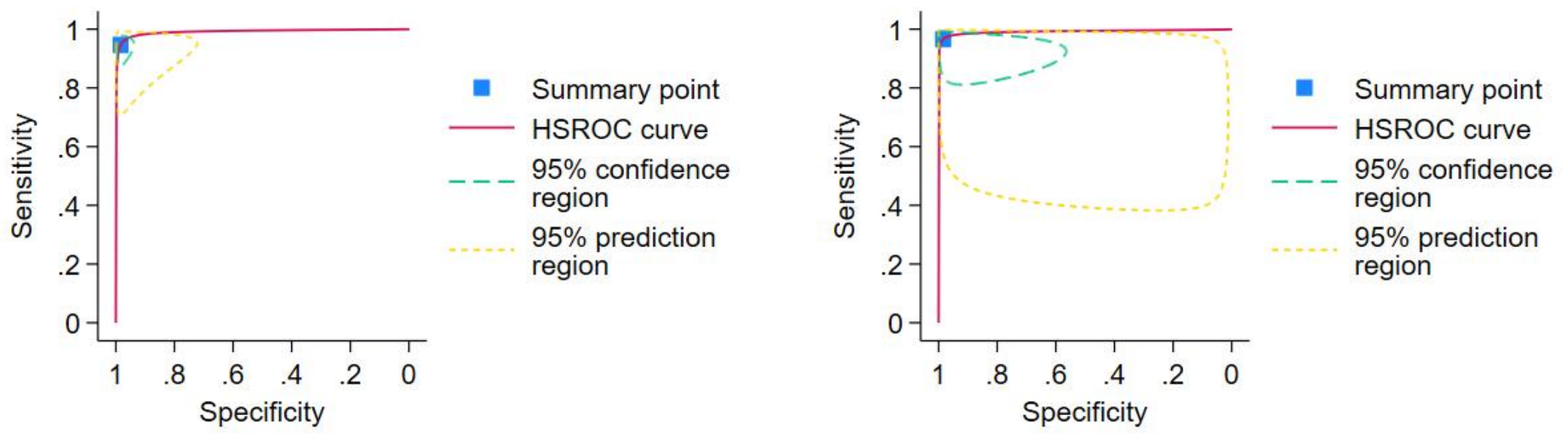

Fig 5 HSROC curve of input number in ≥median 2662 group and ＜2662 group
